# Kif15 Is Required in the Development of Auditory System Using Zebrafish as a Model

**DOI:** 10.3389/fnmol.2022.844568

**Published:** 2022-03-18

**Authors:** Shimei Zheng, Dongmei Tang, Xin Wang, Chang Liu, Na Zuo, Renchun Yan, Cheng Wu, Jun Ma, Chuanxi Wang, Hongfei Xu, Yingzi He, Dong Liu, Shaofeng Liu

**Affiliations:** ^1^Department of Otolaryngology-Head and Neck Surgery, Yijishan Hospital of Wannan Medical College, Wuhu, China; ^2^State Key Laboratory of Medical Neurobiology and MOE Frontiers Center for Brain Science, ENT Institute and Department of Otorhinolaryngology, Eye & ENT Hospital, Fudan University, Shanghai, China; ^3^NHC Key Laboratory of Hearing Medicine, Fudan University, Shanghai, China; ^4^Nantong Laboratory of Development and Diseases, School of Life Sciences, Co-innovation Center of Neuroregeneration, Key Laboratory of Neuroregeneration of Jiangsu and MOE, Nantong University, Nantong, China; ^5^Department of Forensic Medicine, Soochow University, Suzhou, China

**Keywords:** Kif15, morpholino knockdown, auditory organs, development, zebrafish

## Abstract

Kif15, a kinesin family member, is powerful in the formation of bipolar spindles. There is emerging evidence indicating that Kif15 plays vital roles in influencing the growth of axons and interference with the progression of the tumor. However, the function of Kif15 in the auditory organs remains unknown. The Western blotting test was used to examine the effect of Kif15 downregulation by specific morpholino targeting Kif15 (Kif15-MO). The development of the inner ear and posterior lateral line (PLL) system in zebrafish was under continuous observation from spawns to 96 h postfertilization (hpf) to investigate the potential role of Kif15 in the auditory and vestibular system. We uncovered that Kif15 inhibition induced otic organ deformities in zebrafish, including malformed semicircular canals, abnormal number and location of otoliths, and reduced number of hair cells (HCs) both in utricle and saccule. Furthermore, a remarkable reduction in the number of PLL neuromasts was also explored in Kif15-MO morphants compared to the normal larvae. We also detected notably reduced activity in locomotion after Kif15 was knocked down. Additionally, we performed rescue experiments with co-injection of Kif15 mRNA and found that the Kif15 splicing MO-induced deformities in otic vesicle and PLL of zebrafish were successfully rescued, and the severely reduced locomotor activity caused by Kif15-MO was partially rescued compared to the control-MO (Con-MO) embryos. Our findings uncover that Kif15 is essential in the early development of auditory and vestibular organs using zebrafish as models.

## Introduction

Kif15 (also called Kinesin-12), a pivotal member of the kinesin family, is essential for the production of bipolar spindles (Sturgill and Ohi, 2013). Recent studies have revealed that some kinesin family members are exploited for the invention of antimitotic drugs especially used in cancer therapy (Milic et al., 2018). Among those, Eg5 (Kif11 or Kinesin-5) and Kif15 are most highlighted. Eg5 is fundamental for spindle assembly while Kif15 is non-essential in the establishment of spindle bipolarity when Eg5 is in full activity. However, Kif15 becomes essential in the maintenance of spindle bipolarity when Eg5 is partially inhibited (Tanenbaum et al., 2009). Kif15 is also demonstrated involved in developing neurons, influencing axonal growth, navigation, and branching. For example, using morpholino (MO) injection strategy or CRISPR/Cas9-based knockout technology, the axons grow faster and longer when the Kif15 level is reduced compared to the Kif15 wild type (Liu et al., 2010; Dong et al., 2019). More and more studies show that Kif15 plays a pretty important role in the occurrence and development of some cancers (Gao et al., 2020a,b; Kitagawa et al., 2020; Ma et al., 2020). Moreover, previous studies have demonstrated that Kif15 also plays a pretty important role in mitosis, meiosis, spermiogenesis, cell growth, and differentiation (Ma et al., 2017; Malaby et al., 2019; Yu et al., 2019). Increasingly, the functions of Kif15 in distinct models have gained more attention. As reported by Mei Liu’s laboratory, Kif15 is detected from two-cell stage, mainly concentrated in the central nervous system from 14 to 30 hpf and also distributed in organs such as brain, eyes, ears, fin, and olfactory bulbs in zebrafish (Xu et al., 2014). However, the role of Kif15 in ears has not been explored.

In recent years, zebrafish has become a popular experimental model in the study of auditory system development and inner ear hair cell (HC) regeneration for advantages in the transparent body, short growth period, large oviposition, direct observation, and easier regulation (Blanco-Sánchez et al., 2017; He et al., 2017; Tang et al., 2019, 2021). The mechanosensory organs of zebrafish are composed of the otic vesicle, anterior lateral line in the cephalic region, and posterior lateral line (PLL; Kimmel et al., 1995). Different from the mammals, zebrafish is devoid of the cochlea, and the main components of zebrafish otic vesicle are three pairs of semicircular canals, two pairs of sensory patches named utricle and saccule (Kimmel et al., 1995; Whitfield et al., 2002; Geng et al., 2013). Otoliths composed of calcium carbonate crystalline are overlain on the two maculae; thus, utricle and saccule are called otolith organs (Whitfield et al., 2002; Lundberg et al., 2015; Kalka et al., 2019). The PLL neuromasts (NMs) consist of HCs in the center and supporting cells and mantle cells in the periphery; these HCs share similar structure and function with those in the mammalian inner ear (Whitfield et al., 2002; Nicolson, 2017).

In this study, we first investigated the function of Kif15 in the development of zebrafish otic vesicle and PLL using splicing- and translation-blocking MO targeting Kif15. Our findings uncover the pivotal role of Kinesin superfamily member Kif15 in the development of hearing organs for the purpose of hearing research.

## Materials and Methods

### Zebrafish Maintenance and Operation

The embryos of zebrafish were obtained from *Tg(cldnb:lynGFP)^zf^*^106^ line, *Tg(brn3c:mGFP)^s^*^356^*^t^* line, and AB wild type. Spawns were maintained in embryo medium at 28.5°C according to the standard recipe. Hours/days postfertilization (hpf/dpf) was used to take a record of marking embryos’ different developmental stages. Embryos lived in E3 water which add 0.03‰ 1-phenyl-2-thiourea (PTU; Sigma–Aldrich, St Louis, MO, United States) at the stage of 14 hpf, in the result of avoiding pigment formation. The Institutional Animal Care and Use Committee of Fudan University approved all the animal experiments.

### Morpholino Injections

Two endogenous blocking strategies were used for inhibiting the expression of Kif15 (Kif15-MO), and the precise sequences were shown as follows:

Splicing-blocking: 5′-ATGTATTAAAAACCTCACCTGG CTG-3′;
Translation-blocking: 5′-CATGATTCATTACTATATTTC CTCT-3′.

A total final concentration of 0.3 mM was injected into the yolk sacs of zebrafish embryos at the one- to two-cell stage for Kif15 knockdown. As a control, the parallel embryos were injected with the standard control-MO (Con-MO), sequenced in 5′-CCTCTTACCTCAGTTACAATTTATA-3′ to avoid errors due to injections.

### Imaging

The live zebrafish were anesthetized in 0.02% MS-222 (ethyl 3-aminobenzoate methanesulfonate; Sigma–Aldrich, Inc., Saint Louis, MO, United States) for 3 min and then placed on the glass slide with E3 water under the corresponding microscope. The images were taken with a stereomicroscope or an inverted fluorescence microscope.

### Zebrafish Locomotion Assay

After treatment, larvae were moved into wells of a 48-well plate (1 fish/well) to monitor their swimming behavior. Zebrafish behavior was monitored by a digital video tracking system (DVTS, Noldus Information Technology, United States). Each larva was allowed to habituate to the test environment of the system for 30 min before the start of the data acquisition. Notably, 10 acoustic/vibrational stimuli (Danio Vision intensity setting 6) with a 20-s interstimulus interval were set and applied. The total swimming distance of the larvae that responded to this stimulus was recorded. Swimming behavior studies were repeated at least three times.

### Western Blotting Analysis

Zebrafish embryos at 48 hpf after removal of yolk sac were immersed in precooled radioimmunoprecipitation assay (RIPA) lysis buffer plus cocktail and phenylmethanesulfonyl fluoride (PMSF) for schizolysis. To know the exact concentrations of the extracted proteins, we chose a BCA Protein Kit (Beyotime Institute Biotechnology, China) with the supernatant of the RIPA lysate after dissolving the samples. After electrophoresis on SDS–PAGE, the separated proteins were transferred onto poly(vinylidene fluoride) (PVDF) membranes (Immobilon-P; Millipore, Bedford, United States). Before incubation with the primary antibodies, the membranes were blocked with 5% skim milk for 2 h at room temperature. Anti-Kif15 (1:400 dilution, Proteintech, Shanghai, China) and anti-glyceraldehyde 3-phosphate dehydrogenase (anti-GAPDH; 1: 2000) were incubated with the membranes for a whole night at 4°C. Corresponding proteins were combined with Goat Anti-Mouse/Anti-Rabbit immunoglobulin G [IgG; (H + L)] HRP (1:2000 dilution, Abways Technology, Shanghai, China) after being incubated for 2 h. Using an ECL Kit (Millipore, United States), such protein bands could be colored and detected by an Azure C280 imager. The membranes need to be washed thoroughly with Tris-buffered saline with Tween 20 (TBST) before each incubation step. All experiments were strictly from three repeats. GAPDH served as the internal reference.

### Statistical Analysis

GraphPad Prism (version, 8.0) supports whole statistical analyses. Comparisons between every two groups were demonstrated with a *t*-test (two-tailed), while multiple comparisons were illustrated using one-way ANOVA. Statistics were all displayed as mean ± standard error of the mean (SEM), with *p*-value < 0.05 identified as statistical significance.

## Results

### Knockdown of Kif15 Inhibits the Normal Formation of Vestibular Apparatus

**FIGURE 1:**
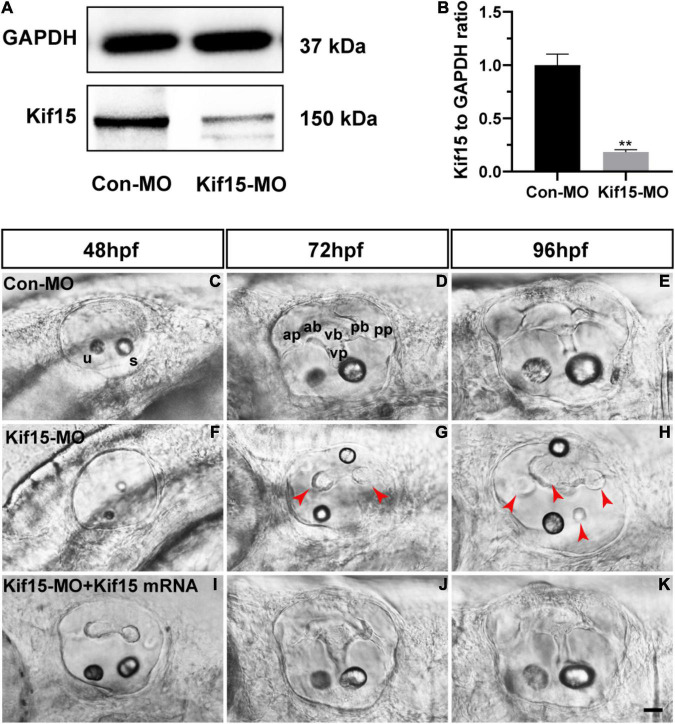
Knockdown of Kif15 with splicing-blocking method inhibits the normal formation of vestibular. **(A,B)** The protein level of Kif15 is severely decreased in Kif15-morpholino (MO)-injected embryos both in the band intensity **(A)** and in the semiquantitative analysis **(B)**, in comparison with control embryos. Data are recorded as mean ± SEM. **p < 0.01. **(C–H)** Kif15-MO suppresses the normal development of semicircular canals. The abnormal manifestations are observed at 48 hpf **(C,F)**, 72 hpf **(D,G)**, and 96 hpf **(E,H)**, respectively. ap, anterior protrusion; ab, anterior bulge; vb, ventral bulge; vp, ventral protrusion; pb, posterior bulge; pp, posterior protrusion; u, utricle; and s, saccule. Red arrowheads show the abnormal fusion of anterior and posterior protrusions. **(I–K)** Co-injection using Kif15-MO and Kif15 mRNA successfully rescues the phenotypes of semicircular canals and otoliths at 48 hpf **(I)**, 72 hpf **(J)**, and 96 hpf **(K)**, respectively. Scale bar is 20 μm.

The role of Kif15 during inner ear development was investigated by the knockdown of Kif15 through the injection of Kif15 splicing MO to the zebrafish embryos at one- or two-cell stages. We used the Western blotting analysis to test the efficacy of Kif15 downregulation, and we detected a substantial transcript reduction of Kif15 in the zebrafish larvae after Kif15-MO injection compared to the controls injected with Con-MO (Figures 1A,B). We chose 48, 72, and 96 hpf in chronology to observe the morphology changes in the inner ear of zebrafish after Kif15-MO injection (Figures 1C–H). At 72 and 96 hpf, the essential structures of three pairs of semicircular canals (i.e., anterior, posterior, and lateral) were clearly discernible as labeled in Figure 1D, including anterior (a-), posterior (p-), and ventral (v-) protrusions and bulges. However, the normal structures of semicircular canals disappeared and were replaced by short and fused protuberances in Kif15-MO morphants (Figures 1F–H). To verify the role of Kif15 on zebrafish semicircular canal development, we performed a rescue experiment by the co-injection of Kif15 mRNA together with Kif15 splicing MO, and we found a successful rescue in the phenotype of otic vesicle (Figures 1I–K). Our observation implied that the inhibition of Kif15 by specific MO completely ruined the normal development of semicircular canals in the inner ear of zebrafish.

### Kif15 Inhibition Induces Malformed Otolith Organs in Zebrafish

**FIGURE 2:**
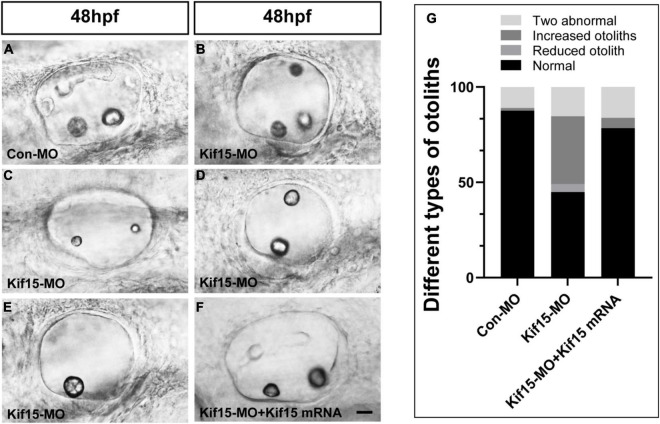
Knockdown of Kif15 disturbs the normal development of otolith organs. **(A–E)** Kif15 inhibition causes abnormal phenotypes of otoliths, including multiplication **(B)** and reduction **(E)** in number, and two abnormal otoliths **(C,D)** at 48 hpf. **(F)** Co-injection Kif15 mRNA with Kif15-MO successfully rescues the phenotypes of otolith organs at 48 hpf. **(G)** Different types of abnormal otoliths and the corresponding proportion analysis in Kif15-MO-injected embryos (*n* = 234), untreated controls (*n* = 136), and Kif15-MO + Kif15 mRNA group (*n* = 111). Scale bar is 20 μm.

The morphology of otoliths in control and Kif15-MO-injected embryos was detected with a white optical microscope in the bright field at 48 hpf (Figures 2A–E). Kif15 splicing MO-injected embryos exhibited various abnormalities containing increased number of otoliths (35.5%), decreased number of otoliths (4.3%), and two abnormal otoliths (15.4%) (Figures 2B–E,G). Among them, the two abnormal otolith deformities involved situations such as the abnormal size of two otoliths (Figure 2C), the abnormal arranged position of two otoliths (Figure 2D), and the combined disordered form. The proportion of normal otolith phenotype in the Kif15-MO group is only 44.9% in contrast to 87.5% in controls (Figure 2G). On the contrary, there is almost no abnormality in the number of otoliths in the Con-MO injection group (Figure 2G). We also detected successful rescue in the phenotype of otoliths after co-injecting Kif15 mRNA and Kif15 splicing MO compared to that in Kif15-MO morphants (Figures 2F,G).

### Both Utricular and Saccular HCs Decreased Severely After Kif15 Knockdown

**FIGURE 3:**
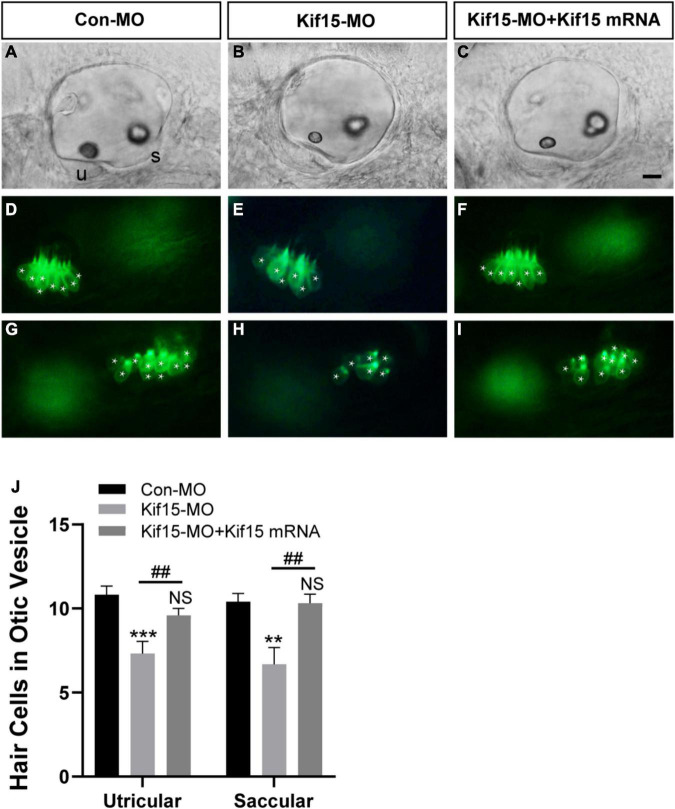
Kif15 inhibition results in decreased hair cells (HCs) in the inner ear. **(A–C)** The gross morphology of otic vesicle in different groups at 48 hpf using the white light field microscope. u, utricle; s, saccule. Scale bar is 20 μm. **(D–F)** Representative images of utricular HCs in different groups at 48 hpf. **(G–I)** Representative images of saccular HCs in different groups at 48 hpf. White star labels the HC. **(J)** Quantification of the number of HCs in utricle and saccule in control-MO (Con-MO) group (*n* = 22), Kif15-deficient morphants (*n* = 22), and Kif15-MO + Kif15 mRNA group. Data are recorded as mean ± SEM. **p < 0.01, ***p < 0.001 vs. Con-MO group; ^##^*p* < 0.01 vs. Kif15-MO group; NS, no significance.

Using *Tg(brn3c:mGFP)^s^*^356^*^t^* line, the HCs that insert into the otoliths were identifiable and labeled by green fluorescence, named utricular (anterior to left) and saccular (posterior to right) HCs, respectively (Figures 3A,D,G). After Kif15 knockdown with splicing MO, two smaller sizes of otoliths were examined, and the numbers of both utricular and saccular HCs in otic vesicles were reduced significantly compared to the controls (Figures 3B,E,H). However, the decreased number of HCs in both utricle and saccule after Kif15 knocking down could be completely rescued by co-injection with Kif15 mRNA (Figures 3C,F,I). The quantitative analysis further showed that the number of utricular HCs in Kif15 morphants was significantly decreased at 48 hpf compared to that in Con-MO-injected larvae, while a remarkably increased number of utricular HCs were found in Kif15-MO + Kif15 mRNA group (Figure 3J). Similarly, HCs in saccule also severely reduced in Kif15-MO-injected morphants but markedly increased in Kif15-MO + Kif15 mRNA co-injected embryos (Figure 3J).

### Kif15-MO Leads to Decreased Number of Neuromasts in Posterior Lateral Line System of Zebrafish

**FIGURE 4:**
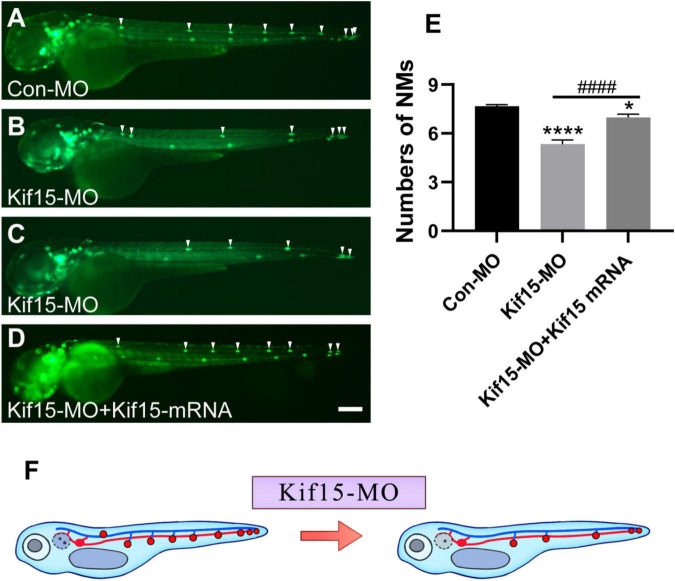
Kif15 knocking down leads to abnormal posterior lateral line (PLL) development, which is represented by a decrease in the number of neuromasts (NMs). **(A–D)** Kif15 inhibition induces the reduction of NMs in zebrafish PLL, while co-injection of Kif15 mRNA with Kif15-MO successfully rescues the decreased number of NMs compared to the Con-MO-treated controls. The PLL NMs are indicated by white arrowheads. Scale bar is 200 μm. **(E)** Statistical analysis in quantification of PLL NMs in Con-MO-injected controls, Kif15-MO-injected embryos, and Kif15-MO + Kif15 mRNA group (*n* = 50 in the three groups). Data are recorded as mean ± SEM. *p < 0.05, ****p < 0.0001 vs. Con-MO group; ^####^*p* < 0.0001 vs. Kif15-MO group. **(F)** Simple and intuitive schematic diagram of changes in PLL development after Kif15 knockdown by MO.

Then, the function of Kif15 during the development of the PLL system was investigated. The transgenic zebrafish *Tg(cldnb:lynGFP)^zf106^* was used to visualize NMs in green fluorescence (Haas and Gilmour, 2006). The NMs manifested in severely reduced number and disordered arrangement along the trunk of zebrafish after Kif15-MO injection at 48 hpf, a time point when PLL primordium finishes migration (Figures 4A–C). Co-injecting Kif15 mRNA and splicing MO notably increased the number of NMs in comparison with that in Kif15-MO morphants (Figure 4D). The quantitative analysis showed that the number of PLL NMs in Kif15 morphants was significantly decreased compared to that displayed in Con-MO-injected larvae at 48 hpf (Figure 4E). The number of PLL NMs in the Kif15-MO + Kif15 mRNA group was remarkably larger than that in Kif15-MO morphants but a little smaller than that in Con-MO embryos, indicating a successful but not complete rescue in the number of NMs by Kif15 mRNA (Figure 4E). The diagrammatic sketch demonstrating changes in the number and arrangement of NMs along the trunk and tail of zebrafish was presented after injection with Kif15-MO (Figure 4F). The result provided substantial evidence that Kif15 is critical to zebrafish in auditory system development.

### Kif15 Transcript Knockdown Disrupts the Normal Locomotor Activity of Zebrafish

**FIGURE 5:**
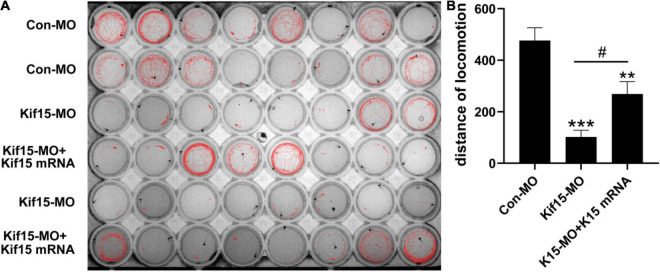
The activity and behavior of zebrafish are abnormal after knocking down Kif15. **(A)** The locomotion assay of zebrafish in Con-MO (*n* = 32), Kif15-MO (*n* = 31), and Kif15-MO + Kif15 mRNA (*n* = 30) injected larvae in traced locomotion curve. **(B)** The moved distance from the original point is analyzed and compared among different groups. The unit of the Y-axis is mm. Data are recorded as mean ± SEM. **p < 0.01, ***p < 0.001 vs. Con-MO group; *^#^p* < 0.05 vs. Kif15-MO group.

According to the brief review of the literature in the background that sensory organs of zebrafish including otoliths, semicircular canals, and PLL NMs can detect sound and water movement stimuli and move in directionality, we wondered if the locomotor behavior of zebrafish was disturbed in Kif15-MO embryos. The locomotion assay was conducted to detect the locomotor behavior of zebrafish after Kif15 inhibition with splicing MO since behavioral change is a comprehensive indicator to show the impact of environmental factors on the organisms. The locomotor traces were recorded during the light-dark photoperiod stimulation with a 20-s interstimulus interval, and we detected a severe reduction in locomotor traces compared to that in the controls (Figure 5A). However, a significant increase in locomotor traces was found after performing the rescue experiment with co-injected Kif15 mRNA compared to that in Kif15-MO morphants (Figure 5A). Data analysis on the distance moved from the original point in different groups showed that the Kif15-MO injection caused a significant reduction in the distance of locomotion in comparison with the parallel controls. There was also a significant difference in distance of movement between Kif15-MO + Kif15 mRNA and Con-MO groups, indicating a partial rescue effect of Kif15 mRNA in the locomotor behavior (Figure 5B). The results were indicative that behavioral dysfunction also existed in addition to morphological deformity after Kif15 inhibition.

## Discussion

Kif15 has been previously reported to be widely involved in the progression of several malignancies, such as hepatocellular carcinoma (HCC), and gastric, breast, colorectal, and prostate cancer by regulating cell cycle, migration, and invasion (Li et al., 2020; Zeng et al., 2020; Qureshi et al., 2021). The previous study detected high expression of Kinesin-12 (Kif15) in the otic vesicle of zebrafish (Xu et al., 2014), indicating a potential role of Kif15 in the embryonic development of the inner ear. However, to our knowledge, there are no reports considering the effect of Kif15 on the auditory system. In this study, we first explored the role of Kif15 in the developing hearing organ of zebrafish using the knocking down strategy by Kif15-MO injection.

An MO knockdown approach is a popular tool in studying the function of objective genes (Moulton and Yan, 2008; Stainier et al., 2017); however, the known off-target effect by MOs limits the application of MO to some extent (Gerety and Wilkinson, 2011). In this study, we chose splicing-blocking MO for Kif15 knockdown, and the efficacy of Kif15 inhibition in transcript level was examined by the Western blotting experiment with anti-Kif15 antibody. We found that the formations of otolith organs, semicircular canals, and PLL NMs were all remarkably deformed in the structure after the inhibition of Kif15. Besides, the further rescue experiments with Kif15 mRNA demonstrated successful rescue of the phenotypical deformities caused by Kif15-MO. To confirm our findings by Kif15 splicing MO, we performed an experiment using the translation-blocking MO strategy for Kif15. As shown in Supplementary Figure 1, the expression of Kif15 was significantly decreased in Kif15 translation MO morphants compared to the Con-MO group (Supplementary Figures 1A,B). Additionally, malformed otoliths and semicircular canals, together with the reduced number of PLL NMs, were also found in Kif15 translation MO morphants (Supplementary Figures 1C,D), which were consistent with the findings in Kif15 splicing MO morphants. Therefore, our findings uncovered that Kif15 is essential in the maintenance of the normal structures and functions of auditory organs of zebrafish, and Kif15-MO with splicing- and translation-blocking strategy was potent for the regulation of Kif15.

As previously reported, the knockdown of Kif15 inhibits cell proliferation, promotes cell apoptosis, and causes cell cycle arrest in glioma cells (Wang et al., 2020). Another study reveals that the inhibition of Kif15 in human HCC xenograft models delays the invasive and proliferative ability of tumors *via* increasing intracellular reactive oxygen species (ROS) levels (Li et al., 2020). In this study, Kif15-MO induced remarkable inhibition of the HC differentiation in both utricle and saccule. Since the proliferative behavior is active in the early development of zebrafish inner ear and lateral line system, we speculated that the reduced number of NMs in PLL and decreased HCs in otic vesicle might be related to the suppression of cell proliferation or increased apoptosis by Kif15 knockdown. Moreover, we detected abnormal locomotion in the Kif15-MO morphants, and the reduced locomotor distance could be partially rescued by co-injection with Kif15 mRNA, indicating a potential role of Kif15 in zebrafish mechanosensory organ function. However, Kif15 is also known to influence neuronal development so it is possible that these defects are simply locomotor defects unrelated to the response to the vibrational stimulus (Xu et al., 2014; Dong et al., 2019). Therefore, in future study, we will use alternative approaches such as prepulse assay to test if abnormal locomotion by Kif15 knockdown is due to ear defects. Altogether, we explored that Kif15 plays an important role during the development of the auditory and vestibular system of zebrafish.

## Data Availability Statement

The original contributions presented in the study are included in the article/Supplementary Material, further inquiries can be directed to the corresponding author/s.

## Ethics Statement

The animal study was reviewed and approved by The Institutional Animal Care and Use Committee of Fudan University approved all the animal experiments.

## Author Contributions

YH, DL, and SL: conceptualization, methodology, writing—review and editing, and project administration. SZ, DT, CL, XW, NZ, RY, CWu, HX, JM, and CWa: methodology and formal analysis. SZ, DT, and XW: validation, investigation, and formal analysis. All authors read and approved the final manuscript.

## Conflict of Interest

The authors declare that the research was conducted in the absence of any commercial or financial relationships that could be construed as a potential conflict of interest.

## Publisher’s Note

All claims expressed in this article are solely those of the authors and do not necessarily represent those of their affiliated organizations, or those of the publisher, the editors and the reviewers. Any product that may be evaluated in this article, or claim that may be made by its manufacturer, is not guaranteed or endorsed by the publisher.

## Supplementary Material

Supplementary Figure 1 Knockdown of Kif15 with the translation-blocking method inhibits the normal development of zebrafish. **(A,B)** The protein level of Kif15 is severely decreased in Kif15-MO (translation-blocking)-injected embryos both in the band intensity **(A)** and in the semiquantitative analysis **(B)**, compared to the control embryos. Data are recorded as mean ± SEM. ^***^*p* < 0.001. **(C)** Kif15-MO (translation-blocking) disturbs the normal development of otic vesicles. The abnormal manifestations in both otolith organs and semicircular canals are observed at 48, 72, and 96 hpf, respectively. Scale bar is 20 μm. **(D)** A reduced number of NMs in zebrafish PLL is detected in Kif15 morphants (translation-blocking). Scale bar is 200 μm, *n* = 10.
