# Supplementary figures and images for: Kif15 Is Required in the Development of Auditory System Using Zebrafish as a Model
Source: Front Mol Neurosci. 2022 Mar 18;15:844568. doi: 10.3389/fnmol.2022.844568 (PMC8971910; doi:10.3389/fnmol.2022.844568)

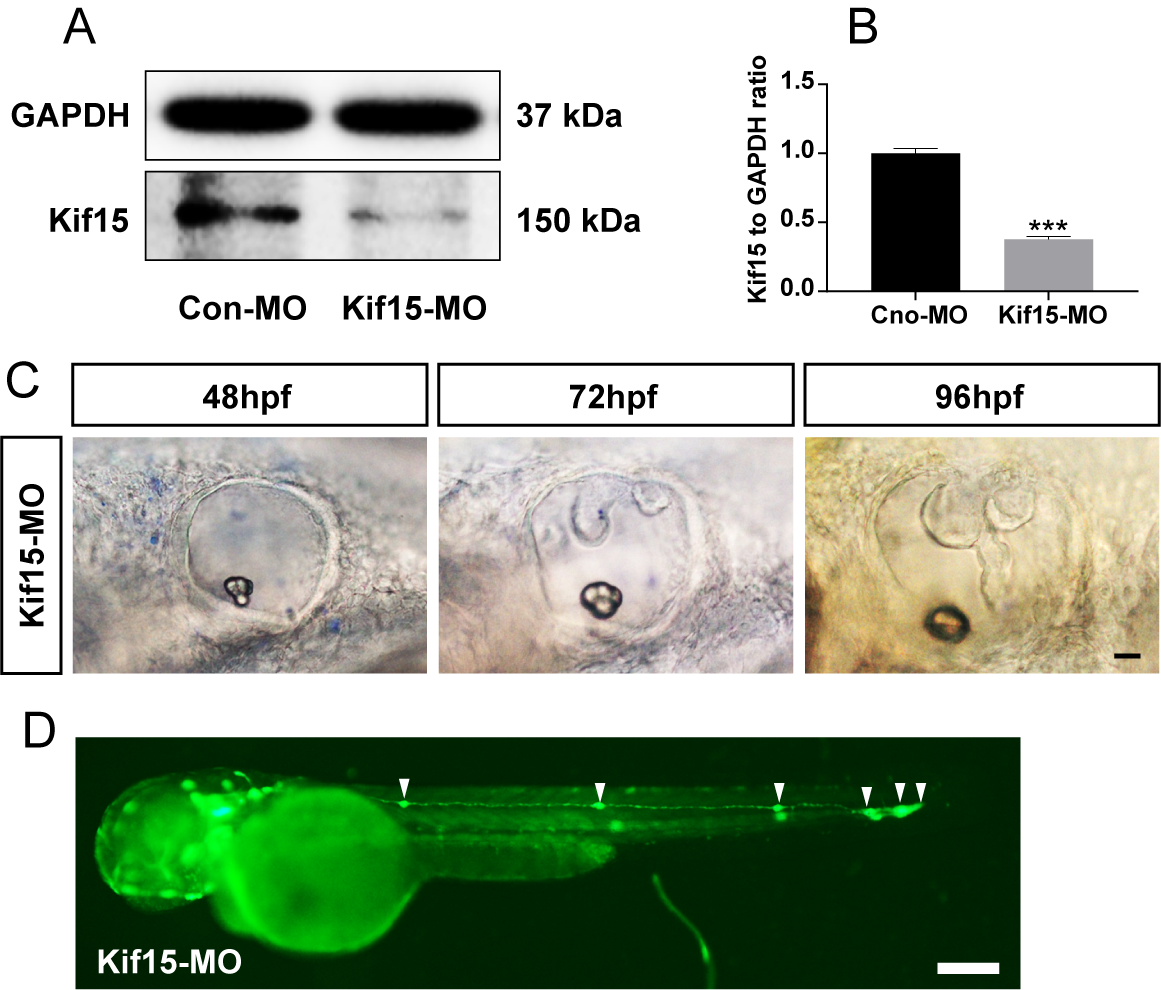

Supplement: Supplementary Figure 1 — Knockdown of Kif15 with the translation-blocking method inhibits the normal development of zebrafish. (A,B) The protein level of Kif15 is severely decreased in Kif15-MO (translation-blocking)-injected embryos both in the band intensity (A) and in the semiquantitative analysis (B), compared to the control embryos. Data are recorded as mean ± SEM. ***p < 0.001. (C) Kif15-MO (translation-blocking) disturbs the normal development of otic vesicles. The abnormal manifestations in both otolith organs and semicircular canals are observed at 48, 72, and 96 hpf, respectively. Scale bar is 20 μm. (D) A reduced number of NMs in zebrafish PLL is detected in Kif15 morphants (translation-blocking). Scale bar is 200 μm, n = 10. [file Image_1.tif]
